# METTL3 knockout accelerates hepatocarcinogenesis via inhibiting endoplasmic reticulum stress response

**DOI:** 10.1002/2211-5463.70023

**Published:** 2025-03-18

**Authors:** Bo Cui, Silin Tu, Haibo Li, Zhancheng Zeng, Ruiqi Xiao, Jing Guo, Xiaoqi Liang, Chang Liu, Lijie Pan, Wenjie Chen, Mian Ge, Xiaofen Zhong, Linsen Ye, Huaxin Chen, Qi Zhang, Yan Xu

**Affiliations:** ^1^ Biotherapy Centre, the Third Affiliated Hospital Sun Yat‐sen University Guangzhou China; ^2^ Department of Hepatic Surgery and Liver Transplantation Center the Third Affiliated Hospital of Sun Yat‐sen University Guangzhou China; ^3^ Cell‐Gene Therapy Translational Medicine Research Centre, the Third Affiliated Hospital Sun Yat‐sen University Guangzhou China; ^4^ Laboratory Animal Center Sun Yat‐sen University Guangzhou China; ^5^ Guangdong Provincial Key Laboratory of Liver Disease Research, the Third Affiliated Hospital Sun Yat‐sen University Guangzhou China; ^6^ Department of Anesthesiology, the Third Affiliated Hospital Sun Yat‐sen University Guangzhou China

**Keywords:** ER stress response, hepatocarcinogenesis, hepatocellular carcinoma, m^6^A, METTL3

## Abstract

Hepatocellular carcinoma (HCC) is among the most common causes of cancer‐related deaths worldwide. Previous studies showed that N6‐methyladenosine (m^6^A), the most abundant chemical modification in eukaryotic RNAs, is implicated in HCC progression. Using liver‐specific conditional knockout mice, we found that the loss of METTL3, the core catalytic subunit of m^6^A methyltransferase, significantly promoted hepatic tumor initiation under various oncogenic challenges, contrary to the previously reported oncogenic role of METTL3 in liver cancer cell lines or xenograft models. Mechanistically, we hypothesized that METTL3 deficiency accelerated HCC initiation by inhibiting m^6^A deposition on *MANF* transcripts, impairing nuclear export and thus MANF protein levels, which led to insufficient endoplasmic reticulum (ER) stress response pathway activation. Our findings suggest a tumor‐suppressive role for METTL3 in the early stages of HCC, emphasizing the importance of understanding the dynamic role of epigenetic regulation in tumorigenesis and targeted therapy.

## Graphical abstract

Liver‐specific knockout of N^6^‐methyladenosine (m^6^A) methyltransferase METTL3 significantly accelerated hepatic tumor initiation under various oncogenic challenges, contrary to the previously reported oncogenic role of METTL3 in liver cancer cell lines or xenograft models. Mechanistically, METTL3 deficiency reduced m^6^A deposition on *Manf* transcripts and impaired *Manf* mRNA nuclear export and expression, thereby inhibiting the endoplasmic reticulum (ER) stress response during hepatocarcinogenesis.

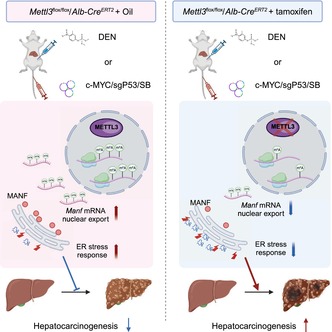

**Abbreviations**
ALT: alanine transaminase
AST: aspartate transaminase
DBIL: direct bilirubin
DEGs: differentially expressed genes
DEN: diethylnitrosamine
ER stress: endoplasmic reticulum stress
FBS: fetal bovine serum
GSEA: gene set enrichment analysis
H&E: hematoxylin–eosin
HCC: hepatocellular carcinoma
HTVI: hydrodynamic tail vein injection
IHC: immunohistochemistry
LDH: lactate dehydrogenase
m^6^A: N6‐methyladenosine
MANF: mesencephalic astrocyte‐derived neurotrophic factor
METTL14: methyltransferase 14
METTL3: methyltransferase 3
TBIL: total bilirubin
UPR: unfolded protein response
WTAP: Wilm's tumor 1‐associating protein

Hepatocellular carcinoma (HCC) is the most common type of primary liver cancer, accounting for approximately 75–85% of all cases [1]. The majority of HCC originates against a backdrop of chronic liver disease, including conditions such as viral hepatitis, alcoholic liver disease, and metabolic‐associated fatty liver disease [2, 3]. Under the persistent or repeated stimulus of inflammation and fibrosis, alterations in the hepatic genome, epigenomics, and gene expression patterns occur, gradually accumulating and ultimately leading to tumor formation [4, 5, 6]. Despite advances in medical and surgical interventions, HCC remains a substantial challenge worldwide due to its late diagnosis and rapid disease progression [7]. Most patients are diagnosed at advanced stages, making them ineligible for potentially curative treatments such as surgical resection [8]. This grim scenario underscores the urgent need for early diagnosis strategies and innovative therapeutic targets that can improve the overall outcomes of patients with HCC.

N6‐methyladenosine (m^6^A), the most abundant reversible modification found in eukaryotic mRNAs, regulates various aspects of RNA metabolism, including stability, nuclear export, alternative splicing, and translation, thereby influencing RNA fate and subsequent gene expression [9]. The dynamic regulation of m^6^A methylation is mediated by three key groups of proteins: methyltransferases (referred to as “writers”), demethylases (“erasers”), and m^6^A‐binding proteins (“readers”) [10]. Methyltransferase 3 (METTL3) is the key regulator of m^6^A methylation deposition, forming the “writer” complex with methyltransferase 14 (METTL14) and Wilm's tumor 1‐associating protein (WTAP) [11]. It has been shown that METTL3 is elevated in many cancer types (including liver cancer) and associated with poor prognosis [12]. However, previous research has explored the role of METTL3 mainly in established HCC using cancer cell lines or xenograft models, which could not fully recapitulate the early stages of malignant transformation [13]. This knowledge gap highlights the need to elucidate the function of METTL3 in early hepatocarcinogenesis.

Here, we dissected the role of METTL3 and its mediated m^6^A modification during the early phases of HCC development. By employing liver‐specific METTL3 inducible knockout mice, we explored how the loss of METTL3 influenced hepatocyte transformation and the onset of HCC. Our findings suggest that METTL3 and m^6^A modification play a tumor‐suppressive role in the early stages of HCC, challenging the existing paradigm of its function based on studies in established cancer models. This research could pave the way for novel diagnostic and therapeutic strategies targeting m^6^A modification pathways in early HCC.

## Materials and methods

### Human specimens

HCC and paired adjacent nontumor specimens used in this study were obtained from patients at the Third Affiliated Hospital of Sun Yat‐sen University. All specimens were embedded in paraffin for sectioning and subsequent immunohistochemical evaluation. This study was approved by the Human Ethics Committee of the Third Affiliated Hospital of Sun Yat‐sen University, with the ethics approval number RG2024‐207‐01. Written informed consent was secured from all patients involved in the study. The whole study conformed to the ethical guidelines of the 1975 Declaration of Helsinki.

### Mice

Control and hepatocyte‐specific METTL3 inducible knockout mice (*Mettl3* cKO) were generated and maintained as previously described [14]. Male mice at 6–8 weeks old were used for experiments. All mice were housed in a specific pathogen‐free facility under a 12‐h light/dark cycle with access to food and water *ad libitum*. Mice were anesthetized with a ketamine and xylazine mixture at the experimental endpoint, followed by liver portal vein perfusion and tissue collection for analysis. All experimental procedures were approved by the Institutional Animal Care and Use Committee (IACUC) of the Third Affiliated Hospital of Sun Yat‐sen University, with the ethics approval number IACUC‐F3‐22‐0220.

For hydrodynamic tail vein injection (HTVI) HCC model, the plasmid mix used in this experiment included 20 μg of pX330‐U6‐sgTP53‐CBh‐hsp‐cas9 (sg*P53*), 20 μg of pT3‐EF1a‐c‐MYC (c‐MYC), and 1.6 μg of pPGK‐SB13, all of which were kindly provided by SM. Zhuang [15]. The plasmid mixture was dissolved in 2 mL of saline solution and rapidly injected into the mouse tail vein within 7 s, as previously described [16]. Samples were collected at 3 or 5 weeks post injection.

For the diethylnitrosamine (DEN)‐induced liver cancer model, DEN (N0756; Sigma‐Aldrich, St. Louis, MO, USA) was prepared in saline at 2.5 mg·mL^−1^. Both control and *Mettl3* cKO mice received an intraperitoneal injection of DEN (10 μL·g^−1^) on Day 15 after birth. At 4 weeks old, mice were injected intraperitoneally with either Oil or Tam to induce hepatocyte‐specific knockout of METTL3. Tissue samples were harvested 9 months after DEN treatment.

### Biochemical analysis

Mice serum samples were collected by centrifuging at 3824 *g* for 10 min. The serum levels of aspartate transaminase (AST), alanine transaminase (ALT), lactate dehydrogenase (LDH), total bilirubin (TBIL), and direct bilirubin (DBIL) were detected using a Hitachi 7020 automatic biochemical analyzer (Hitachi, Tokyo, Japan).

### Western blot and immunohistochemistry

Western blot and immunohistochemistry (IHC) were conducted as previously described [17]. Quantification of the positive area of METTL3 and KI67 was performed with the tissuefaxs software (Tissue Gnostics, Vienna, Austria) using protocols that automatically detected the tissue and manually delineated the positive area by visual inspection. The antibodies are listed in Table S1.

### Real‐time quantitative reverse transcription PCR (RT‐qPCR)

RNA extraction, reverse transcription, and RT‐qPCR were performed as previously described [17]. The primers used are listed in Table S2.

### Aggresome detection

The misfolded and/or aggregated proteins were detected in frozen liver sections using the Proteostat Aggresome Detection Kit (ENZ‐51035; ENZO, New York, NY, USA) according to the manufacturer's instructions. In brief, frozen liver tissues were sliced into 8‐μm‐thick sections and permeabilized. Liver sections were blocked and incubated with the Aggresome Kit for 30 min, and cell nuclei were counterstained with Hoechst 33342 (C1022; Beyotime, Shanghai, China). Fluorescent images were captured using the laser confocal microscope (LSM 980; Zeiss, Oberkochen, Germany). Quantification of the aggresome was conducted with three random fields for each individual.

### RNA‐sequencing and m^6^A‐RIP sequencing

RNA‐sequencing was conducted by Berry Genomics (Beijing, China), and m^6^A‐RIP sequencing was conducted by Guangzhou Epibiotek Co., Ltd. (Guangzhou, China). In short, for RNA‐sequencing, total RNA was isolated from liver tissues of control and *Mettl3* cKO mice 3 weeks post‐HTVI induction (four samples per group). The sequencing reads were aligned to the mouse genome version mm10 using bowtie2 [18] and rsem [19], and differentially expressed genes (DEGs) were identified using deseq2 [20]. For m^6^A‐RIP sequencing, liver tissues were collected from 8‐week‐old control mice (two replicates), and mRNA was extracted, fragmented, and enriched by m^6^A antibody. The sequencing reads were aligned to the mouse genome version mm10 with hisat2 [21]. m^6^A peaks were identified and analyzed using the exomepeak r package [22]. RNA‐sequencing and m^6^A‐RIP sequencing data are available in the Gene Expression Omnibus (GEO) database under the accession numbers GSE289320 and GSE198511, respectively.

### Primary hepatocyte isolation

Mouse primary hepatocytes were isolated from mice aged 6–8 weeks by two‐step collagenase perfusion methods. In brief, mice were euthanized and perfused through portal vein cannulation by EGTA buffer (8000 mg·L^−1^ NaCl, 400 mg·L^−1^ KCl, 76.67 mg·L^−1^ NaH_2_PO_4_, 120.45 mg·L^−1^ Na_2_HPO_4_, 2380 mg·L^−1^ HEPES, 350 mg·L^−1^ NaHCO_3_, 190 mg·L^−1^ EGTA, and 900 mg·L^−1^ Glucose, pH 7.35–7.4) at a pump rate of 8 mL·min^−1^ for 3 min, followed by enzyme buffer containing 100 U·mL^−1^ collagenase IV (C5138; Sigma‐Aldrich, St. Louis, MO, USA) at the same pump rate for another 8 min. The EGTA buffer and enzyme buffer were prewarmed in a water bath at 42 °C. The cell suspension was then filtered through a 70 μm cell strainer (251200; Sorfa, ZJ, China). The filtered cells were collected by centrifugation at 50 ***g*** for 1 min at 4 °C. After centrifugation, the cells were resuspended in DMEM‐high glucose medium (C11995500BT; Thermo Scientific, Waltham, MA, USA) supplemented with 10% fetal bovine serum (FBS) (P30‐3302; PAN, Aidenbach, Germany) and 1% penicillin/streptomycin (KGY0023; KeyGEN Biotech, JS, China) and seeded in Type I Collagen (354236; Corning, NY, USA) precoated culture plates. The cell viability was measured by the trypan blue exclusion assay. Two hours post‐seeding, the medium was replaced to remove unattached cells. An additional 4 hours later, the culture medium was further replaced with William's E Medium (12551032; Gibco, Grand Island, NY, USA) with 1% penicillin/streptomycin for further treatment.

### mRNA stability of mouse primary hepatocytes

Primary hepatocytes were isolated from control and *Mettl3* cKO mice as previously described [17] and allowed to adhere for 6 hours before adding 5 μg·mL^−1^ actinomycin D (A1410; Sigma‐Aldrich, St. Louis, MO, USA). Samples were collected at indicated time points post‐treatment, followed by the extraction of cellular RNA for RT‐qPCR analysis.

### m^6^A‐RIP qPCR

m^6^A‐RIP qPCR was performed using mouse liver tissues 3 weeks after HTVI induction as previously described [23]. Total RNA was extracted, and Poly (A) mRNAs were purified using the GenEluteTM mRNA Miniprep Kit (MRN10; Sigma‐Aldrich, St. Louis, MO, USA). The mRNA was fragmented and incubated with anti‐m^6^A antibody or mouse IgG (as negative control) for 2 hours at 4 °C. Protein A and Protein G beads were added to the mixture and incubated for another 2 hours at 4 °C. RNase inhibitor (N2611; Promega, Madison, WI, USA) was added throughout the entire process. The mRNA was eluted and extracted using the phenol–chloroform method. The precipitated mRNAs were reverse transcribed, and their enrichment was determined by RT‐qPCR. Primers used for m^6^A‐RIP qPCR are listed in Table S3.

### Statistical analysis

Results are expressed as mean ± standard error of the mean (SEM). The Student's *t*‐test was used to compare the two groups of data. A one‐way analysis of variance followed by Dunnett's test was used to compare data from three or more groups. *P* < 0.05 was considered statistically significant.

## Results

### METTL3 is upregulated in HCC and is associated with poor prognosis

**Fig. 1:**
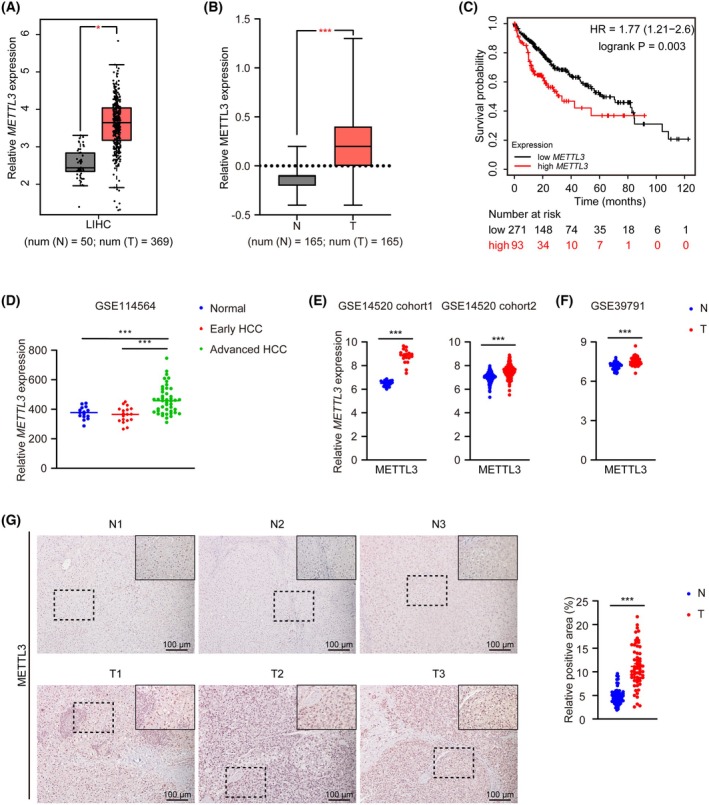
METTL3 is upregulated in HCC and associated with poor prognosis. (A) The mRNA expression level of *METTL3* in liver cancer and adjacent normal tissues from the TCGA database analyzed by GEPIA (http://gepia.cancer‐pku.cn/). N, adjacent nontumor tissue; T, tumor (also hereafter in similar experiments) (*n* (N) = 50; *n* (T) = 369). (B) The protein expression level of METTL3 in liver cancer and paired adjacent normal tissues. Data were retrieved from The Human Protein Atlas (HPA) database (*n* (N) = 165; *n* (T) = 165). (C) Correlation between METTL3 expression level and overall survival rates of liver cancer patients in the TCGA database (*n* (high) = 93; *n* (low) = 271). (D) mRNA expression of *METTL3* in normal, early HCC, and advanced HCC liver tissues (data were retrieved from GSE114564) (*n* = 15 for normal group; *n* = 18 for early HCC group; *n* = 45 for advanced HCC group). (E, F) mRNA expression profile of *METTL3* in tumor and paired nontumor tissues in GSE14520 (E) (*n* = 19/group for cohort 1; *n* = 214/group for cohort 2) and GSE39791 (F) (*n* = 72/group). (G) Representative brightfield images (left) and quantification (right) of immunohistochemical staining for METTL3 in tumor tissues and paired adjacent nontumor tissues from HCC patients (*n* = 12/group). Cell nuclei were counterstained with hematoxylin (also hereafter in similar experiments). Scale bar = 100 μm. The quantification was conducted with 5 random fields (10*) for each sample. Data in (A), (B), and (D–G) are reported as the mean ± SEM with the indicated significance (*P < 0.05, ***P < 0.001. Student's *t*‐test).

To investigate the role of METTL3 in HCC, we initially analyzed the expression profile of METTL3 in HCC and control liver tissues from data in the Cancer Genome Atlas (TCGA) database and The Human Protein Atlas (HPA) database (tandem mass tag (TMT) mass spectrometry data of paired tumor and adjacent nontumor liver tissues originally from the CPTAC dataset [24]). The results revealed a significant increase in both mRNA and protein levels of METTL3 in HCC tissues compared with adjacent nontumor tissues (Fig. 1A,B). Kaplan–Meier survival analysis showed that HCC patients with higher METTL3 expression exhibited poorer overall survival (Fig. 1C). Additionally, analysis of publicly available GEO datasets (GSE114564) confirmed the upregulation of METTL3 in advanced HCC tumor tissues compared with healthy human livers and early HCC tissues (Fig. 1D). Similarly, analysis of another two GEO datasets of tumor and paired nontumor samples (GSE14520 and GSE39791) also showed that METTL3 expression was significantly higher in HCC tumor tissues than in adjacent nontumor tissues (Fig. 1E,F). Immunohistochemistry staining (IHC) for patient liver specimens further confirmed the markedly higher METTL3 expression in tumor tissues than matched nontumor tissues (Fig. 1G). We also tested the METTL3 expression in a mouse spontaneous HCC model induced by HTVI of the Sleeping Beauty transposon system expressing the oncogene c‐MYC and vectors expressing Cas9 and single‐guide RNA targeting P53 (sg*P53*) [16]. Consistent with the pattern in HCC patients, IHC staining and western blot both showed a dramatic increase in METTL3 protein in liver tissues from tumor‐inducing mice relative to those from controls, with higher levels in tumor regions than in adjacent nontumor regions (Fig. S1A–C). Collectively, these data showed that METTL3 was upregulated during HCC progression and associated with poor prognosis, consistent with previous studies [25].

### Hepatocyte‐specific knockout of METTL3 accelerates tumorigenesis in different hepatocarcinogenesis models

**Fig. 2:**
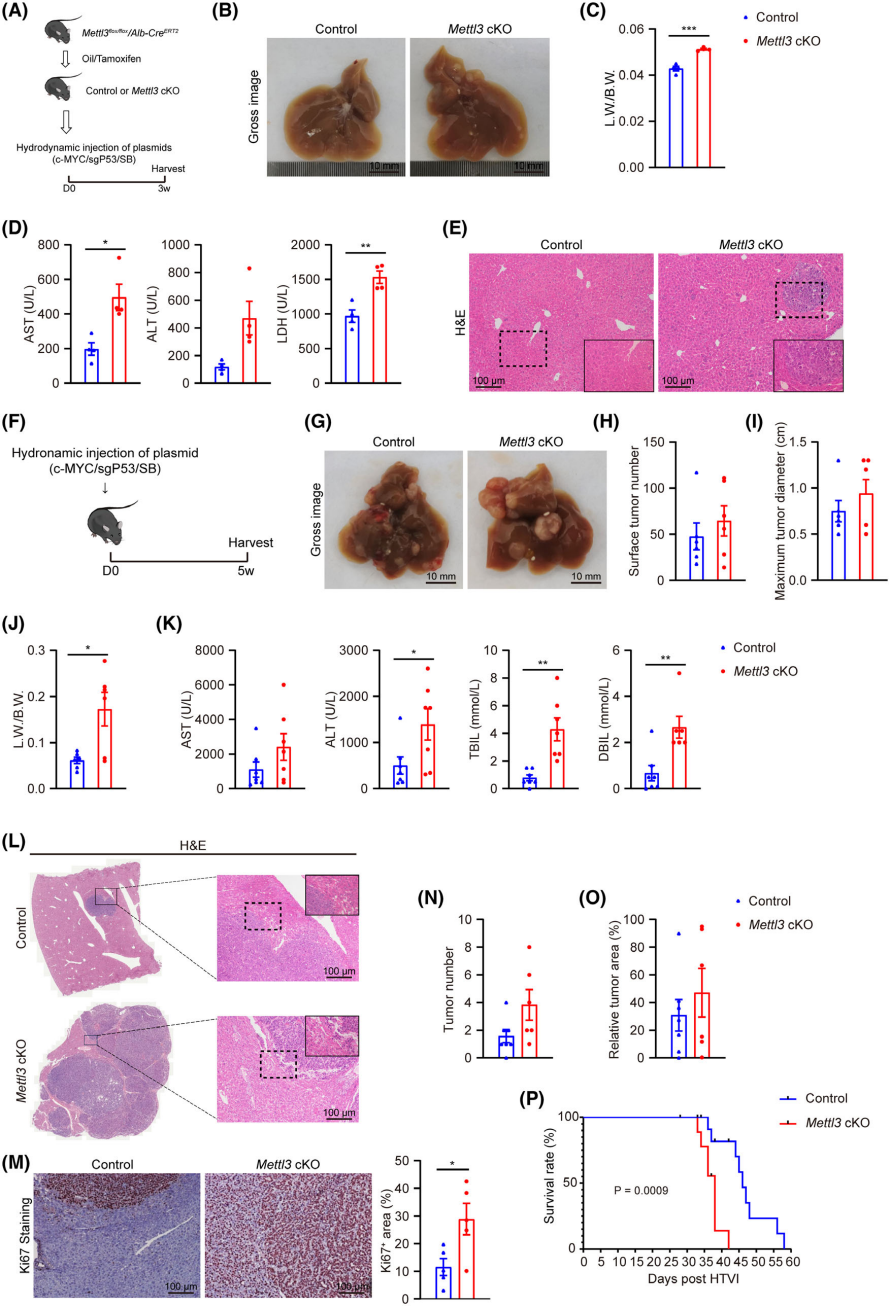
Hepatocyte‐specific knockout of METTL3 accelerates HTVI‐induced hepatocarcinogenesis. (A) Schematic diagram of experimental design. (B) Representative gross images of livers from mice in indicated groups 3 weeks after HTVI (*n* = 4/group). Scale bar = 10 mm. (C) Liver weight/body weight (L.W./B.W.) ratio of mice in indicated groups 3 weeks after HTVI (*n* = 4/group). (D) Serum levels of AST, ALT, and LDH of Control and *Mettl3* cKO mice 3 weeks after HTVI (*n* = 4/group). (E) Representative photographs of H&E staining for liver tissues from Control and *Mettl3* cKO mice 3 weeks after HTVI. Scale bar = 100 μm. (F) Schematic diagram of experimental design. (G–M) Representative gross images of livers (G), surface tumor number (H), maximum surface tumor diameter (I), liver weight/body weight ratio (J), serum levels of AST, ALT, TBIL, and DBIL (K), representative photographs of liver tissue H&E staining (L), and representative photographs and quantification of immunohistochemical staining for KI67 of liver tissues (M) from Control and *Mettl3* cKO mice 5 weeks after HTVI treatment (Control: *n* = 7; *Mettl3* cKO: *n* = 6). Scale bar = 10 mm in (G) and 100 μm in (L) and (M). (N, O) Quantification of tumor number (N) and relative tumor area (O) in H&E staining of liver tissues from Control and *Mettl3* cKO mice 5 weeks after HTVI (Control: *n* = 7; *Mettl3* cKO: *n* = 6). (P) Survival curve of HTVI‐treated mice in indicated groups (Control: *n* = 9; *Mettl3* cKO: *n* = 8). The *P*‐value is for the Long‐rank test. Data in (C), (D), (H–K), and (M–O) are reported as the mean ± SEM with the indicated significance (*P < 0.05, **P < 0.01, ***P < 0.001. Student's *t*‐test).

To further explore the impact of METTL3 and its mediated m^6^A modification on hepatocarcinogenesis, we subjected the transgenic mice we generated previously [14], in which METTL3 could be inducibly deleted in hepatocytes via tamoxifen treatment, to HCC induction by various oncogenic challenges (Fig. S2A). RT‐qPCR and western blot confirmed efficient ablation of METTL3 in tamoxifen‐treated mice (*Mettl3* cKO) compared with the control mice (Fig. S2B–E). First, we subjected the control and *Mettl3* cKO mice to the c‐MYC/sg*P53* HTVI‐induced HCC model, and samples were collected 3 weeks or 5 weeks post‐HTVI (Fig. 2A,F). *Mettl3* cKO mice presented more significant liver damage 3 weeks after HTVI, evidenced by gross liver images (Fig. 2B), increased liver‐to‐body weight ratio (Fig. 2C), and elevated levels of AST, ALT, and LDH compared to mice in the control group (Fig. 2D). Histological analysis also showed that *Mettl3* cKO mice exhibited more tumor‐like nodules and cells with abnormal nuclear morphology (Fig. 2E). Consistently, at 5 weeks post‐HTVI, the livers of the *Mettl3* cKO group exhibited more notable tumor development (with increased surface tumor number and increased maximum surface tumor diameter) and significantly increased liver‐to‐body weight ratio (Fig. 2G–J), as well as dramatic increases in AST, ALT, TBIL, and DBIL levels (Fig. 2K). The results of hematoxylin–eosin (H&E) and KI67 IHC staining also indicated that hepatocyte‐specific knockout of METTL3 accelerated tumorigenesis (Fig. 2L,M). Moreover, we evaluated the tumor burden through microscopic analysis with H&E staining and found that *Mettl3* cKO mice had a substantially greater number of tumors (Fig. 2N) and larger relative tumor areas (Fig. 2O) compared with the control group. Notably, all *Mettl3* cKO mice succumbed within 42 days post‐HTVI, while the control mice survived much longer (Fig. 2P). These results indicate that hepatic‐specific knockout of METTL3 accelerates tumorigenesis in the c‐MYC/sg*P53* HTVI‐induced HCC model.

Then, we used another commonly used spontaneous HCC model, the DEN‐induced hepatocarcinogenesis model, to confirm the protumorigenesis role of METTL3 in HCC development (Fig. S3A). DEN is a potent chemical carcinogen with a strong affinity for the liver, which induces liver cancer by altering the DNA structure within the liver, forming alkyl‐DNA adducts, and inducing chromosomal aberrations and micronuclei [26]. At the age of 9 months, compared with the control mice treated with DEN, DEN‐treated *Mettl3* cKO mice displayed increased tumor nodules and aggravated changes in liver gross appearance, alongside increases in liver‐to‐body weight ratio and larger maximum surface tumor size (Fig. S3B–E). Serum levels of AST, ALT, and LDH also showed an increasing liver injury index in *Mettl3* cKO mice treated with DEN (Fig. S3F), corroborated by histological examination and KI67 IHC staining analysis (Fig. S3G,H).

It is well‐known that the majority of HCC develops on the foundation of liver fibrosis/cirrhosis [27], and the underlying fibrosis/cirrhosis creates a unique microenvironment (TME) contributing to the initiation, progression, and therapeutic response of HCC [28]. Thus, we also performed Picro Sirius Red (PSR) staining analysis in both mouse models to quantify the fibrosis stage. As expected, significantly enhanced collagen deposition was observed in the peritumoral tissues of *Mettl3* cKO mice compared with controls in both models (Fig. S4A,B). These findings in two independent hepatocarcinogenesis models consistently indicate that the loss of METTL3 in hepatocytes accelerates liver tumorigenesis.

### Hepatic METTL3 deletion inhibits the ER stress response during hepatocarcinogenesis

**Fig. 3:**
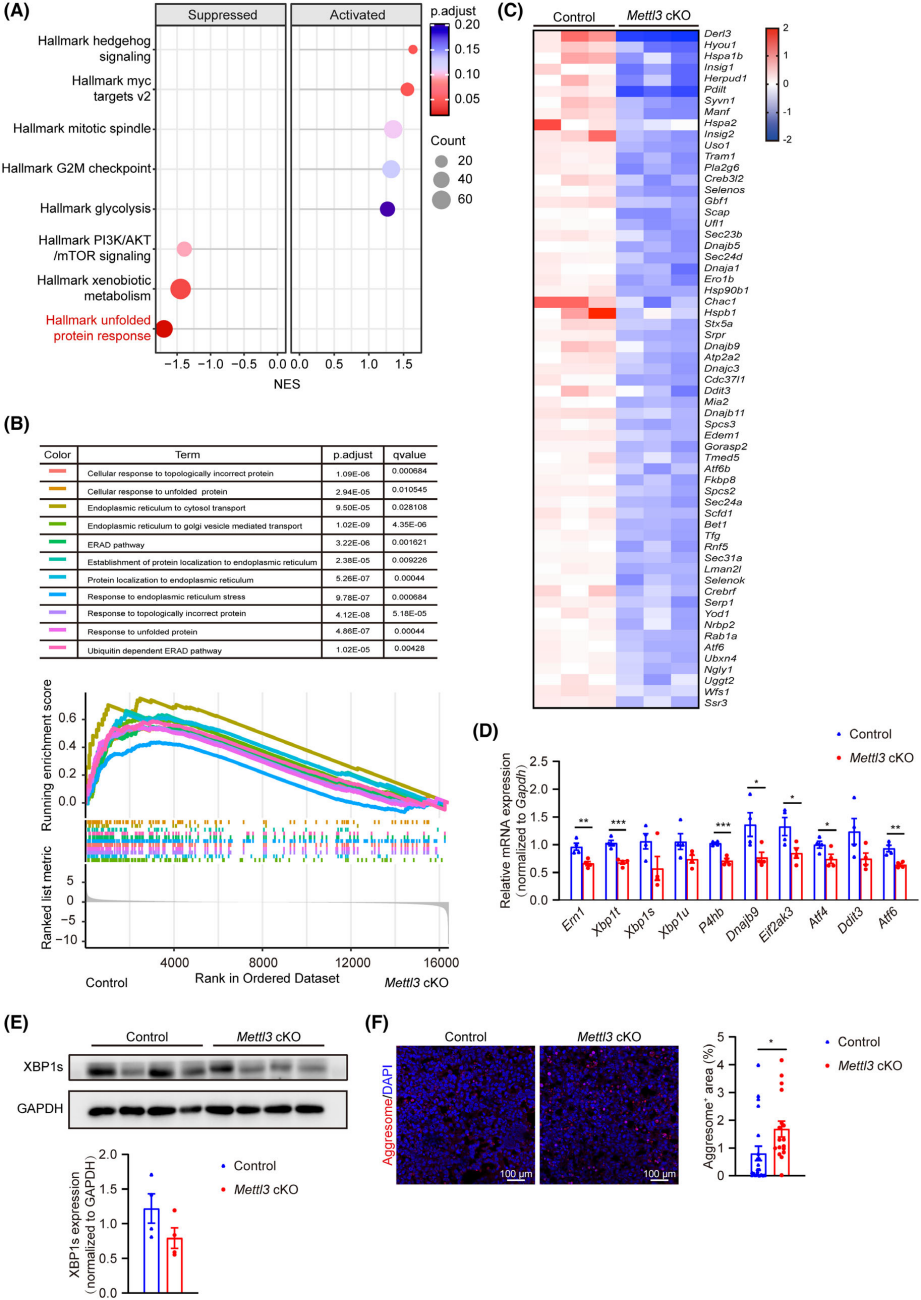
Hepatic METTL3 deletion inhibits ER stress response during hepatocarcinogenesis. (A) Dotplot of GSEA results showing inhibition of ER stress‐related pathways and activation of oncogenic pathways in the *Mettl3* cKO group versus the Control group in RNA‐sequencing data. (B) GSEA plot showing inhibition of ER stress‐related pathways in the *Mettl3* cKO group versus the Control group in RNA‐sequencing data. (C) Heatmap showing expression of genes related to the ER stress response in the Control and *Mettl3* cKO groups from RNA‐sequencing data. (D) RT‐qPCR for key genes in ER stress‐related pathways in liver tissues from Control and *Mettl3* cKO mice 3 weeks after HTVI (*n* = 4/group). *Gapdh* was used as the internal control (also hereafter in similar experiments). (E) Representative western blot and quantification for XBP1s in liver tissues from Control and *Mettl3* cKO mice 3 weeks after HTVI (*n* = 4/group). GAPDH was used as the loading control (also hereafter in similar experiments). (F) Representative fluorescent photographs and quantification of immunofluorescence staining for the aggresome in liver tissues from Control and *Mettl3* cKO mice 5 weeks after HTVI (Control: *n* = 7; *Mettl3* cKO: *n* = 6). Cell nuclei were counterstained with DAPI. The quantification was conducted with three random fields (20*) for each sample. Scale bar = 100 μm. Data in (D–F) are reported as the mean ± SEM with the indicated significance (*P < 0.05, **P < 0.01, ***P < 0.001. Student's test).

To further explore the molecular mechanisms underlying the tumor‐suppressive effects of METTL3 in hepatocarcinogenesis, we conducted RNA‐sequencing on liver tissues from two groups of mice 3 weeks post‐HTVI (four individuals per group) (Fig. S5A). RNA‐sequencing data revealed significant alterations in the transcriptome between the control and *Mettl3* cKO groups. There are 1968 differentially expressed genes in the *Mettl3* cKO group compared with the control group, with the criteria of |fold change| ≥ 1.5 and *P_adj_* < 0.1, with 1252 genes upregulated and 716 genes downregulated. Gene Set Enrichment Analysis (GSEA) showed that MYC targets, mitotic spindle, G2M checkpoint, and glycolysis, all of which are hallmarks of cancer cells [29, 30, 31, 32], were significantly activated in the *Mettl3* cKO group (Fig. 3A), confirming accelerated tumorigenesis after hepatic METTL3 knockout. Interestingly, unfolded protein response (UPR) was the most significantly suppressed pathway within the *Mettl3* cKO group (Fig. 3A). GSEA analysis using gene ontology (GO) of biologic process terms as background also showed significant suppression of endoplasmic reticulum stress (ER stress) response‐related pathways, including unfolded protein response, response to the endoplasmic reticulum, and ER‐associated protein degradation (ERAD) pathway (11 related terms among top 20 enriched terms, Fig. 3B). GO enrichment by either clusterprofiler [33] or metascape [34] and Kyoto Encyclopedia of Genes and Genomes (KEGG) enrichment of downregulated genes further validated inhibition of ER stress‐related pathways in the *Mettl3* cKO group (Fig. S5B–D). Among the 1968 DEGs, 61 genes related to ER stress were significantly downregulated in the *Mettl3* cKO group (Fig. 3C). These results suggest that pathways related to the response to ER stress are significantly inhibited in the early stage of hepatocarcinogenesis of *Mettl3* cKO mice.

ER stress refers to a condition where unfolded or misfolded proteins abnormally accumulate in the ER lumen. It could be caused by increased protein secretion, disrupted ER protein folding, or cellular perturbations, such as lipotoxicity. In response to ER stress, cells either evoke the UPR‐related pathways to orchestrate a series of adaptive mechanisms (including reducing the rate of protein synthesis and translocation into the ER, transactivating ER chaperones and ER secretory genes to facilitate more efficient protein folding, and increasing protein trafficking and elimination of misfolded proteins by autophagy and ERAD pathways) to restore protein folding fidelity and ER homeostasis (“adaptive UPR”), or, conversely, provoke a cell death program when ER stress is overwhelmed and persistent (“maladaptive UPR”) [35, 36]. Evidence showed that ER stress and UPR are activated in HCC development [37, 38]. Moreover, inducing ER stress and UPR may serve as an effective strategy to eliminate transformed cells and thus inhibit tumor progression through UPR‐dependent cell death in HCC [37, 38, 39, 40]. Thus, we did RT‐qPCR to further confirm the expression changes of the three downstream branches of the UPR and ER stress process, including IRE1α, PERK, and ATF6 pathways [41]. The results revealed that the expression of key ER stress response‐related genes was significantly downregulated in liver tissues from the *Mettl3* cKO mice 3 weeks post‐HTVI treatment (Fig. 3D), consistent with RNA sequencing data. Moreover, the protein level of the spliced form of XBP1 (XBP1s), an active transcription factor that plays a vital role in the UPR, was also reduced in liver tissues from HTVI‐treated *Mettl3* cKO mice (Fig. 3E). To further validate the conclusion that UPR pathways were inhibited in METTL3 knockout conditions, we detected the protein aggresomes (inclusion bodies of aggregated, misfolded proteins formed in response to cellular stress) [42] in liver tissues of control and *Mettl3* cKO mice with HTVI‐induced HCC. The results revealed an apparent increase in the presence of protein aggregates in the liver tissues of *Mettl3* cKO mice (Fig. 3F), supporting our speculation that UPR was markedly suppressed in the *Mettl3* cKO group. These results suggested that the loss of METTL3 expression promotes hepatocarcinogenesis by suppressing UPR‐associated gene expression during HCC development.

### METTL3 deficiency inhibited UPR during hepatocarcinogenesis by inhibiting the nuclear export and expression of MANF in an m^6^A‐dependent way

**Fig. 4:**
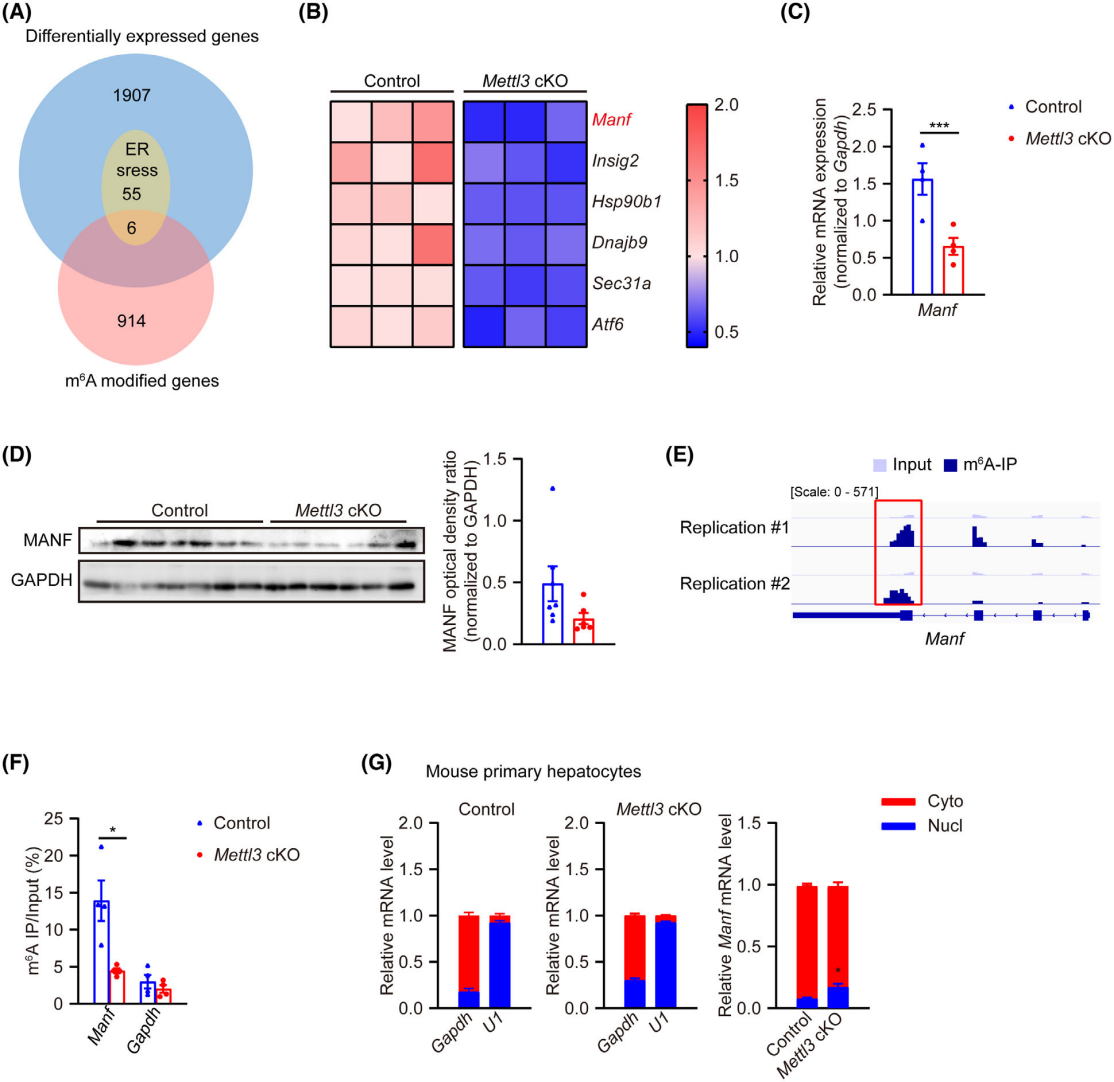
METTL3 deficiency inhibited the nuclear export and expression of MANF in a m^6^A‐dependent way during hepatocarcinogenesis. (A) Venn diagram of differentially expressed genes (DEGs) between mouse liver tissues from the Control and *Mettl3* cKO mice 3 weeks after HTVI, ER stress‐enriched genes in GSEA analysis of RNA sequencing data, and m^6^A‐modified genes in adult liver tissues. (B) Heatmap of overlapping genes in (A). (C) RT‐qPCR for *Manf* in liver tissues from Control and *Mettl3* cKO mice 3 weeks after HTVI (*n* = 4/group). (D) Western blot (left) and quantification (right) for MANF in liver tissues from Control and *Mettl3* cKO mice 5 weeks after HTVI (Control: *n* = 7; *Mettl3* cKO: *n* = 6). (E) Genome Browser screenshots showing read distribution of *Manf* locus in m^6^A‐RIP‐sequencing data of adult mouse liver tissues. (F) m^6^A‐RIP‐qPCR for *Manf* in liver tissues from Control and *Mettl3* cKO mice 3 weeks after HTVI modeling (*n* = 4/group). Positive primer for *Manf* was designed for region within the red box in (E) and *Gapdh* was used as the negative control. (G) RT‐qPCR for *Gapdh* (positive control for cytoplasmic fraction), *U1* (positive control for nuclear fraction), and *Manf* in nuclear (Nucl) and cytoplasmic (Cyto) fractions of mouse primary hepatocytes from Control and *Mettl3* cKO mice (*n* = 3/group). Data in (C), (D), (F), and (G) are reported as the mean ± SEM with the indicated significance (*P < 0.05, ***P < 0.001. Student's *t*‐test).

Next, we conducted m^6^A‐RIP‐sequencing (meRIP‐seq) to identify potential direct targets of METTL3‐mediated m^6^A in adult mouse liver tissues. We found 920 coding genes with significant m^6^A peaks in mouse livers (Fig. 4A). As previously reported [43], m^6^A modification was significantly enriched near the stop codon (Fig. S6A,B). Subsequently, we overlapped differentially expressed genes between mouse liver tissues from the control and *Mettl3* cKO mice 3 weeks after HTVI (1968 genes), ER stress pathway‐enriched genes (61 genes, Fig. 3C), and m^6^A modified genes in adult mouse liver tissues (920 genes) (Fig. 4A). Finally, we got 6 genes that met all the three criteria (Fig. 4A,B). Among them, the expression of mesencephalic astrocyte‐derived neurotrophic factor (MANF) exhibited the most significant change (log_2_ (fold change) = −1.10598714675408, *P*_adj_ = 1.3660938825035E‐08) between the control and *Mettl3* cKO groups (Fig. 4B). MANF, recognized as a central neurotrophic factor, is an endoplasmic reticulum (ER) luminal protein protecting various cells from ER stress [44, 45]. During the progression of HCC, MANF exhibited tumor‐suppressive properties [46, 47]. Thus, we were wondering whether knocking out METTL3 accelerated HCC occurrence by reducing MANF m^6^A modification and expression.

To test our hypothesis, we first did RT‐qPCR and western blot and verified the downregulation of MANF in the *Mettl3* cKO group versus the control group (Fig. 4C,D). meRIP‐seq data revealed a prominent m^6^A modification peak at the 3′ UTR region of the *Manf* transcript, which was also confirmed by meRIP‐qPCR (Fig. 4E,F). Moreover, METTL3 knockout reduced the m^6^A deposition on *Manf* transcripts (Fig. 4F). m^6^A modification affects the stability, nuclear exportation, alternative splicing, translation, and degradation of target mRNAs to be involved in gene expression regulation and, thus, cell fate determination [9]. As both mRNA and protein levels of MANF changed in the *Mettl3* cKO group, we first tested mRNA stability in mouse primary hepatocytes from control and *Mettl3* cKO mice. No significant differences were observed between the two groups (Fig. S6C). Next, we isolated the cytoplasmic and nuclear fractionation of primary hepatocytes from Control and *Mettl3* cKO mice and analyzed the cytoplasmic and nuclear distribution of *Manf* transcripts. The assays demonstrated that the nuclear exportation of *Manf* mRNA was impaired in *Mettl3* cKO hepatocytes (Fig. 4G). These results suggested that METTL3 deficiency promotes hepatocarcinogenesis at least partially by reducing m^6^A modification on *Manf* mRNA, thus impairing the nuclear exportation and subsequent expression of cytoprotective ER stress regulator MANF.

## Discussion

HCC has a poor prognosis and is usually diagnosed only at an advanced stage. METTL3, a key executor of m^6^A methylation modification, has been verified to be involved in the proliferation, invasion, and metastasis of HCC cells, as well as in the glycolysis and lipogenesis processes, promoting the progression of HCC [13]. METTL3 was first identified as an oncogenic factor in HCC due to its upregulation in HCC and subsequent increase in the methylation of the *Suppressor of Cytokine Signaling 2* (*SOCS2*) mRNA, which is then recognized and degraded by YTH Domain‐Containing Family Protein 2 (YTHDF2) [48]. Furthermore, studies have shown that METTL3 is involved in resistance to immune checkpoint inhibitors [49]. Inhibition of METTL3 using the small molecule inhibitor STM2457 enhances the sensitivity of HCC to sorafenib and lenvatinib under hypoxic conditions [50, 51]. Another source of resistance stems from the presence of cancer stem cells, where METTL3 enhances drug resistance through Frizzled Class Receptor 10 (FZD10) in these cells [52]. However, prior studies have primarily investigated the role of METTL3 in established HCC using cancer cell lines or xenograft models. However, these approaches fail to capture the early stages of malignant transformation fully.

In this study, we explored the role and mechanism of METTL3 and its mediated m^6^A modification in the early development of HCC. Contrary to previous findings in established HCC, in our research, METTL3 was identified as a tumor suppressor during the early stage of HCC occurrence. Specifically, knocking out METTL3 in hepatocytes accelerated hepatocarcinogenesis in mouse models of either oncogene‐induced or chemical carcinogen‐induced hepatocarcinogenesis. These seemingly contradictory results indicate that METTL3 plays different roles in established HCC versus early onset stages of HCC. Therefore, our results demonstrate that, unlike its role in established HCC, METTL3 has a tumor‐inhibitory effect on the occurrence of HCC in the early stages of its development, suggesting that future liver cancer treatments targeting METTL3 should consider the stage of tumor progression.

Mechanistically, we found that knocking out METTL3 inhibited ER stress response‐related pathways. The liver plays a central role in secretory and excretory processes. The ER has central functions involving protein synthesis, folding, and transport, as well as lipid synthesis and calcium storage. Sustaining proteostasis is central to maintaining liver function, highlighting the ER as a main node of the proteostasis network during liver homeostasis. However, during various etiologies of HCC development, injury insults resulted in a cellular state known as “ER stress,” which initiates the evolutionarily conserved cell stress response called UPR and ERAD. ER stress contributes to HCC transformation and progression [53, 54, 55, 56, 57, 58]. Moreover, alleviating ER stress prevents hepatocarcinogenesis and HCC progression [38, 55, 59, 60]. Here, we found that METTL3 deficiency accelerates HCC occurrence by inhibiting UPR‐related genes, causing defects in cellular clearance of protein aggregates and prolonged ER stress.

In the meantime, we found that MANF, a secreted endoplasmic reticulum protein protecting various cells from ER stress [44, 45 37, 38], was the most significantly downregulated gene within liver tissues from *Mettl3* cKO mice challenged with oncogenic stimulation. Previous studies showed that mice lacking MANF expression exhibit inflammatory phenotypes and aggravated tissue injury in various tissues, including progressive liver fibrosis and fatty degeneration [61]. In HCC, MANF has also been reported as a tumor suppressor [47]. Our further studies showed that deleting METTL3 reduced m^6^A deposition on *MANF* transcripts and impaired the transport of *MANF* mRNA from the nucleus to the cytoplasm, finally reducing MANF expression in METTL3‐deficient liver tissues. This partially explains our observation that hepatic‐specific METTL3 deficiency showed accelerated HCC occurrence and insufficient ER stress response pathway activation. However, we could not exclude the existence of other possibilities mediating the tumor suppressor function of METTL3 and its mediated m^6^A. For example, during the preparation of our manuscript, two independent groups also reported similar observations with different mechanisms [62, 63]. Wei *et al*. [62] found that METTL3 deletion dramatically accelerates liver tumorigenesis by inducing hepatocyte dedifferentiation and hyperproliferation via m^6^A‐mediated modulation on *Hnf4α* and cell cycle genes. Moreover, the reason for METTL3 induction in HCC also remains to be investigated. Among others, recent studies showed that ER stress activated the METTL3/METTL14 in different contexts [64, 65]. Thus, ER stress and METTL3/METTL14‐mediated m^6^A may form a positive feedback loop to maintain tissue homeostasis, which needs further validation.

In conclusion, our study highlights the suppressive role of METTL3 in the early stages of hepatocellular carcinoma occurrence through its regulation of ER stress pathways. This challenges the traditional view of METTL3 as an oncogene in liver cancer and suggests that its function may be context‐dependent. These insights deepen our understanding of the molecular mechanisms underlying liver cancer and propose new directions for early intervention and therapeutic strategies targeting m^6^A modification pathways.

## Conflict of interest

The authors declare no conflict of interest.

## Author contributions

YX and QZ conceived the idea, provided experimental guidance, analyzed data, and acquired funding. BC, SLT, and HXC contributed to the experimental design and data analysis and conducted most experiments with the help of HBL, ZCZ, RQX, JG, XQL, CL, LJP, WJC, MG, XFZ, and LSY. All authors have read and approved the final version of the manuscript.

## Supporting information


**Fig. S1.** Methyltransferase 3 expression is upregulated in mouse hepatocellular carcinoma tissues.
**Fig. S2.** Construction and characterization of hepatocyte‐specific methyltransferase 3 inducible knockout mice.
**Fig. S3.** Methyltransferase 3 deletion accelerates diethylnitrosamine‐induced liver cancer.
**Fig. S4.** Hepatic methyltransferase 3 knockout aggravates liver fibrosis during hepatocarcinogenesis.
**Fig. S5.** Hepatic methyltransferase 3 knockout inhibits endoplasmic reticulum stress response during hepatocarcinogenesis.
**Fig. S6.** Methyltransferase 3 deficiency inhibited unfolded protein response during hepatocarcinogenesis through mesencephalic astrocyte‐derived neurotrophic factor.
**Table S1.** Antibodies used in the study.
**Table S2.** Sequence of primers for RT‐qPCR.
**Table S3.** Sequence of primers for m^6^A‐RIP‐qPCR.

## Data Availability

The data that support the findings of this study are available in figures and Supporting Information of this article. RNA‐sequencing and m^6^A‐RIP‐sequencing data generated in this study are openly available in NCBI's Gene Expression Omnibus and are accessible through https://www.ncbi.nlm.nih.gov/geo/query/acc.cgi?acc=GSE289320 and https://www.ncbi.nlm.nih.gov/geo/query/acc.cgi?acc=GSE198511, respectively.
