# Supplementary material for: METTL3 knockout accelerates hepatocarcinogenesis via inhibiting endoplasmic reticulum stress response
Source: FEBS Open Bio. 2025 Mar 18;15(7):1144–58. doi: 10.1002/2211-5463.70023 (PMC12226423; doi:10.1002/2211-5463.70023)
Supplement: Supplementary file 1 — Fig. S1. Methyltransferase 3 expression is upregulated in mouse hepatocellular carcinoma tissues. Fig. S2. Construction and characterization of hepatocyte‐specific methyltransferase 3 inducible knockout mice. Fig. S3. Methyltransferase 3 deletion accelerates diethylnitrosamine‐induced liver cancer. Fig. S4. Hepatic methyltransferase 3 knockout aggravates liver fibrosis during hepatocarcinogenesis. Fig. S5. Hepatic methyltransferase 3 knockout inhibits endoplasmic reticulum stress response during hepatocarcinogenesis. Fig. S6. Methyltransferase 3 deficiency inhibited unfolded protein response during hepatocarcinogenesis through mesencephalic astrocyte‐derived neurotrophic factor. Table S1. Antibodies used in the study. Table S2. Sequence of primers for RT‐qPCR. Table S3. Sequence of primers for m6A‐RIP‐qPCR. [file FEB4-15-1144-s001.docx]

**Supplementary Figures**

**
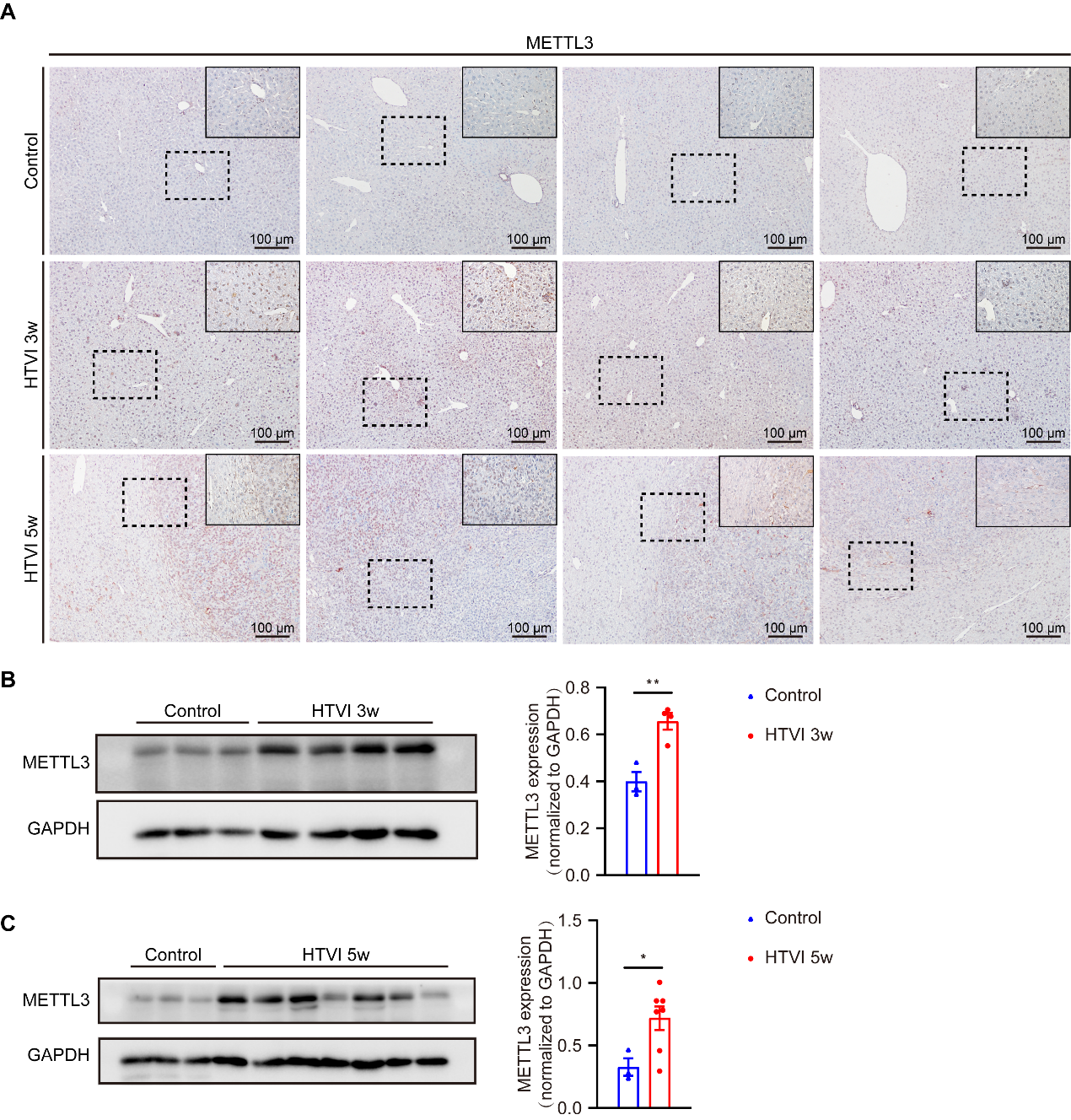
**

**Supplementary Fig. 1. METTL3 expression is upregulated in mouse HCC tissues.** (A) IHC for METTL3 in liver tissues from mice in Control, 3 weeks after HTVI, and 5 weeks after HTVI. Scale bar = 100 μm. (B-C) Western blot and quantitative analysis showing the expression of METTL3 in liver tissues from mice in Control and 3 weeks after HTVI (B) or 5 weeks after HTVI (C) (Control: n = 3; 3 weeks after HTVI: n = 4; 5 weeks after HTVI: n = 7). Data in (B) and (C) are reported as the mean ± SEM with the indicated significance (*p < 0.05, **p < 0.01. Student’s-test).

**
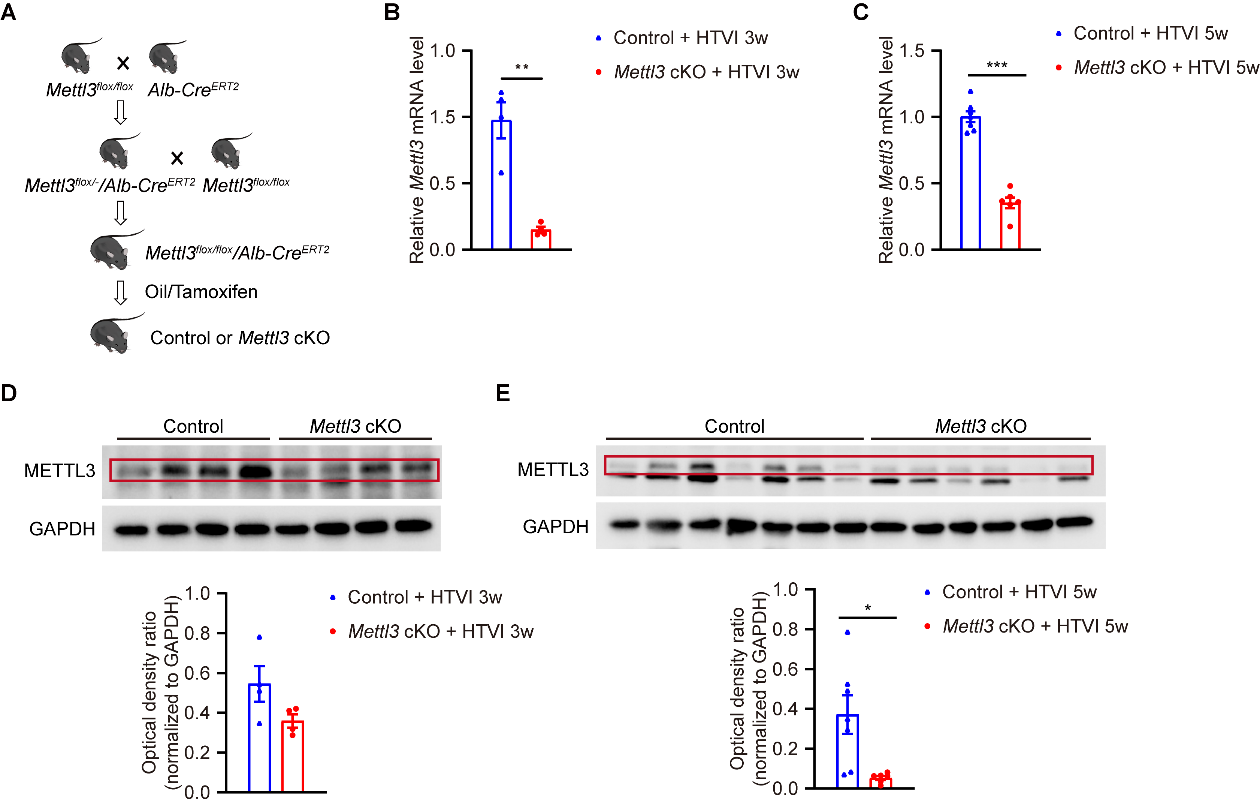
**

**Supplementary Fig. 2. Construction and characterization of hepatocyte-specific METTL3 inducible knockout mice.** (A) Schematic diagram of *Mettl3* cKO mouse construction. (B) RT-qPCR for *Mettl3* of liver tissues from Control and *Mettl3* cKO mice 3 weeks post-HTVI (n = 4/group). (C) RT-qPCR for *Mettl3* in liver tissues from Control and *Mettl3* cKO mice 5 weeks post-HTVI (Control: n = 7; *Mettl3* cKO: n = 6). (D) Western blot and quantification for METTL3 in liver tissues from Control and *Mettl3* cKO mice 3 weeks post-HTVI (n = 4/group). (E) Western blot and quantification for METTL3 in liver tissues from Control and *Mettl3* cKO mice 5 weeks post HTVI (Control: n = 7; *Mettl3* cKO: n = 6). Bands of the expected size were outlined with red boxes in (D) and (E). Data in (B)-(E) are reported as the mean ± SEM with the indicated significance (*p < 0.05, **p < 0.01, ***p < 0.001. Student’s-test).

**
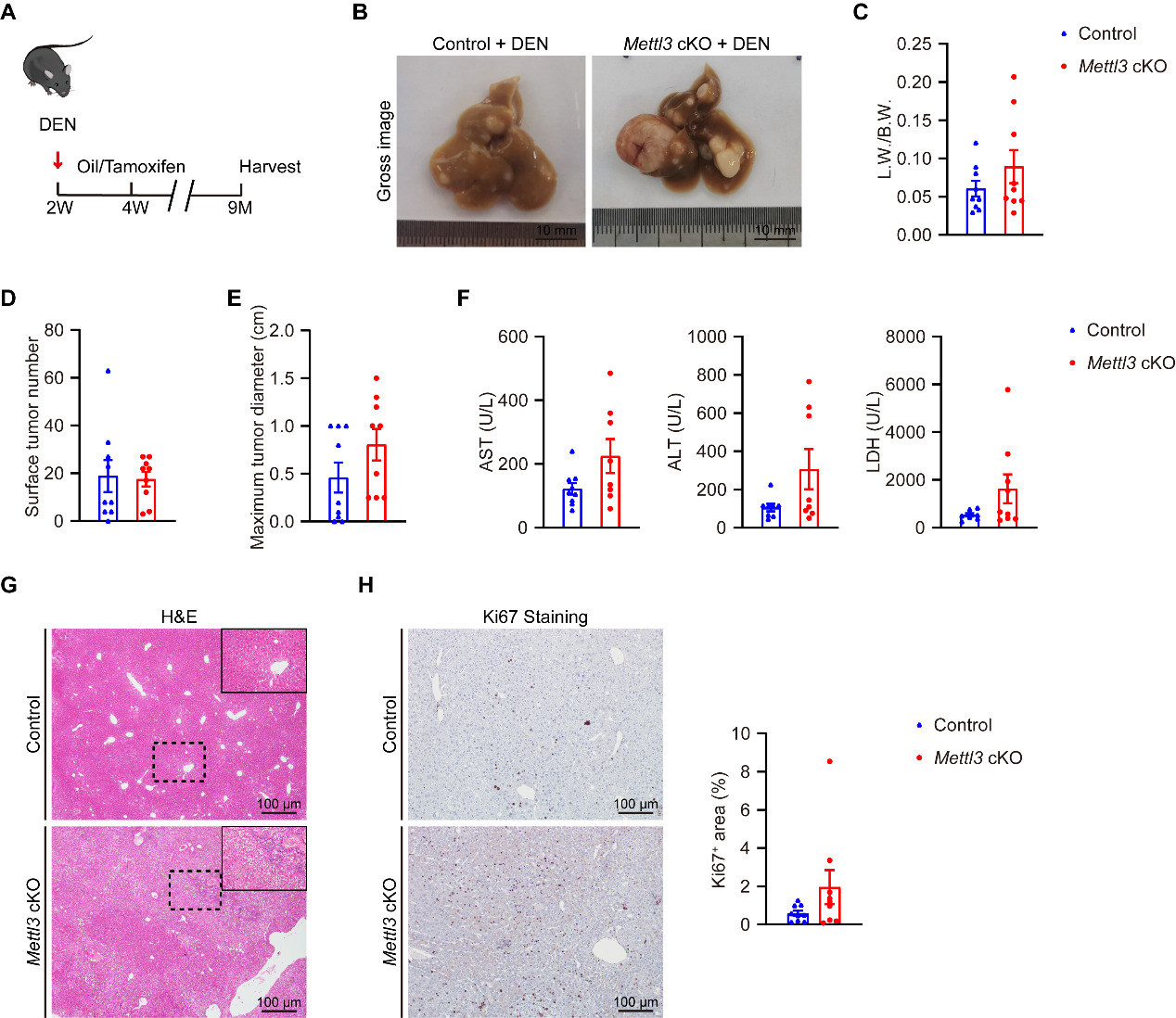
**

**Supplementary Fig. 3. METTL3 deletion accelerates DEN-induced liver cancer.** (A) Schematic diagram of DEN-induced liver cancer model. (B) Representative gross appearance of livers from Control and *Mettl3* cKO group at 9 months. Scale bar = 10 mm. (C) Liver weight/body weight ratio of mice in Control and *Mettl3* cKO group at 9 months old (n = 9/group). (D-E) Surface tumor numbers (D) and maximum tumor diameter (E) in mice of Control and *Mettl3* cKO group at 9 months (n = 9/group). (F) Serum levels of AST, ALT, and LDH in the indicated group of mice at 9 months (n = 9/group). (G) Representative H&E staining images of liver tissues from Control and *Mettl3* cKO mice at 9 months. Scale bar = 100 μm . (H) Representative photographs and quantification of immunohistochemical staining for KI67 of liver tissues from Control and *Mettl3* cKO mice at 9 months (n = 9/group). Scale bar = 100 μm. Data in (C)-(F) and (H) are reported as the mean ± SEM (Student’s-test).

**
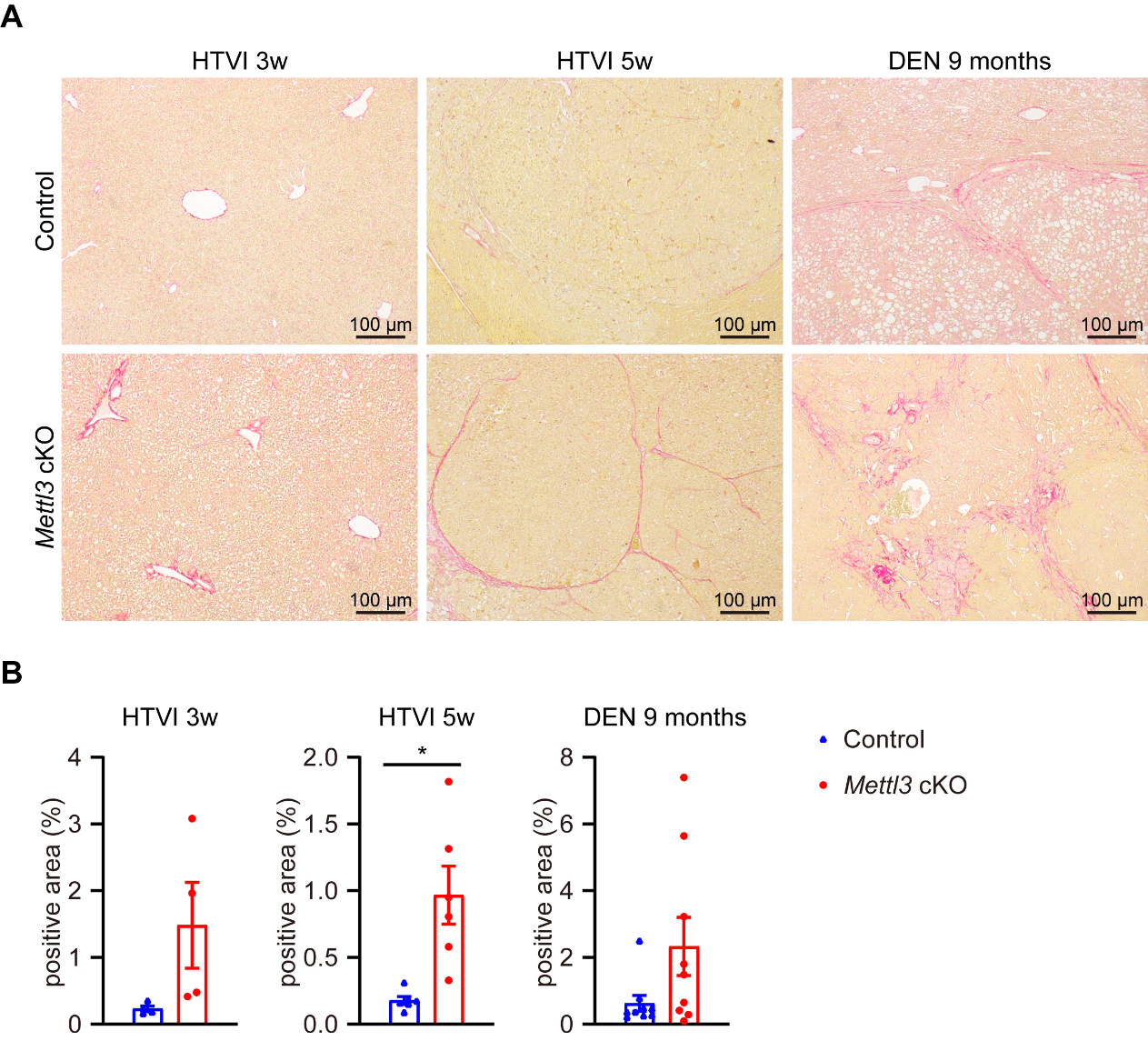
**

**Supplementary Fig. 4. Hepatic METTL3 knockout aggravates liver fibrosis during hepatocarcinogenesis.** (A-B) Representative photographs (A) and quantification (B) of Picrosirius Red (PSR) staining for liver tissues from Control and *Mettl3* cKO mice of 3 weeks or 5 weeks after HTVI and 9 months after DEN groups (HTVI 3 weeks: n = 4/groups; HTVI 5 weeks: Control: n = 7 and *Mettl3* cKO: n = 6; DEN 9 months: n = 9/groups). Scale bar = 100 μm. Data in (B) are reported as the mean ± SEM with the indicated significance (*p < 0.05. Student’s t-test)

**
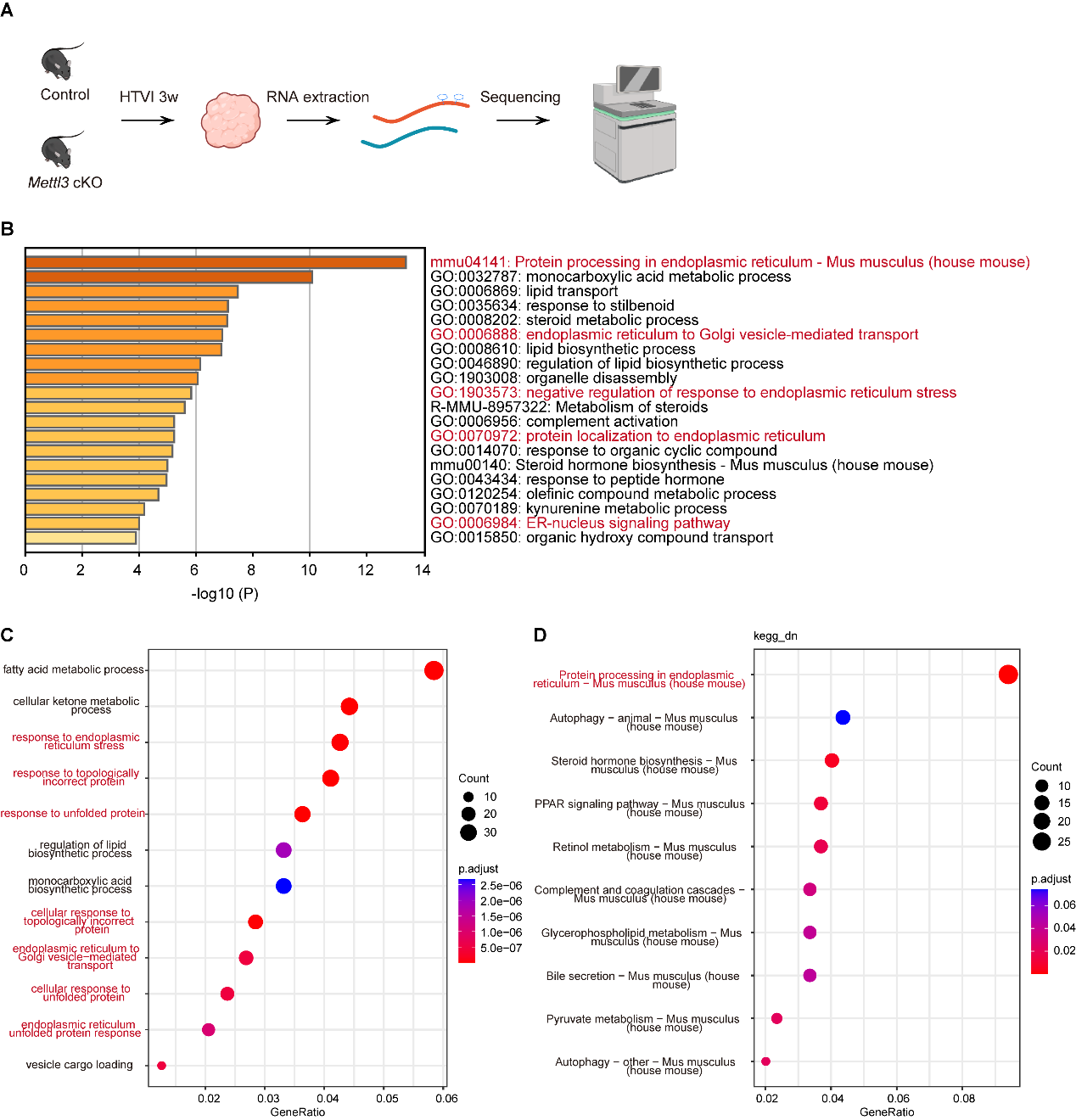
**

**Supplementary Fig. 5. Hepatic METTL3 knockout inhibits ER stress response during hepatocarcinogenesis.** (A) Schematic diagram of experimental design of RNA sequencing. (B) Metascape analysis showing enriched pathways of down-regulated genes in the liver tissues from HTVI-treated (3 weeks) *Mettl3* cKO mice versus Control mice. (C-D) Dotplot showing Gene Ontology (GO) (C) and Kyoto Encyclopedia of Genes and Genomes (KEGG) (D) enrichment analysis (conducted with clusterProfiler) results of down-regulated genes in liver tissues from HTVI-treated *Mettl3* cKO mice versus Control mice.

**
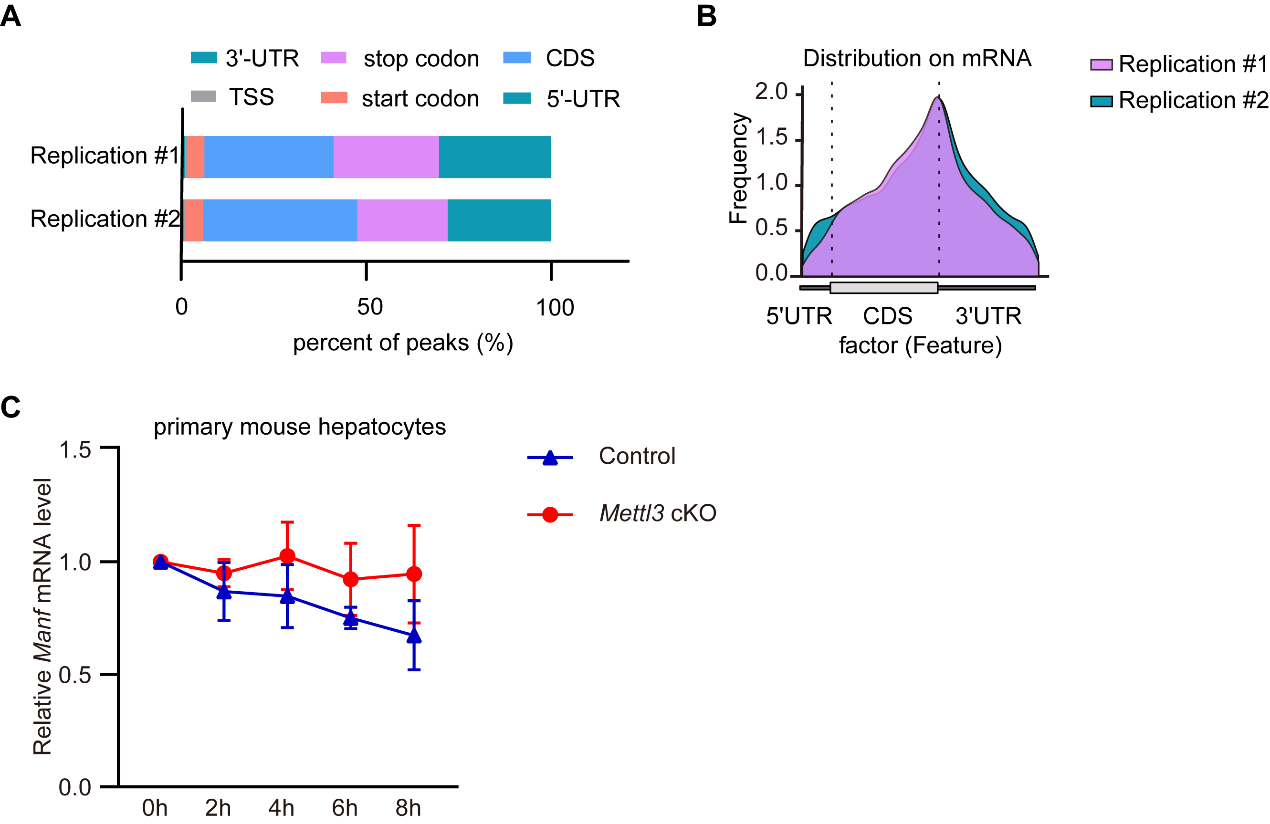
****Supplementary Fig. 6. METTL3 deficiency inhibited UPR during hepatocarcinogenesis through MANF**. (A) Distribution of m^6^A peaks in the 5' UTR, TSS, start codon, CDS region, stop codon, and 3' UTR areas in adult mouse liver tissues. (B) Read distribution of m^6^A-RIP sequencing in adult mouse liver tissues. (C) RT-qPCR for *Manf* in primary hepatocytes isolated from Control and *Mettl3* cKO mice treated with Actinomycin D (5 μg/mL) for the indicated time *in vitro*.

**Supplementary Tables**

***Supplementary Table 1.*** Antibodies used in the study.

| **Name** | **Species reactivity** | **Application and dilution ratio** | **Supplier** | **Cat No.** |
| --- | --- | --- | --- | --- |
| Anti-GAPDH antibody | Mouse, Human | WB (1:1000) | Cell Signaling Technology | Cat# 2118 |
| Anti-METTL3 antibody | Mouse | WB (1:1000) | Servicebio | Cat# GB124688 |
| Anti-MANF antibody | Mouse, Rat, Human | WB (1:1000) | Abcam | Cat# ab67271 |
| Anti-KI67 antibody | Rabbit | IHC (1:200) | Abcam | Cat# ab15580 |
| m^6^A（N6-methyladenosine）antibody | Mouse, Rat, Human | m^6^A-RIP-qPCR | Synaptic Systems | Cat# 202003 |
| Anti-mouse IgG HRP-linked antibody | Mouse | WB (1:5000) | Cell Signaling Technology | Cat# 7076 |
| Anti-rabbit IgG HRP-linked antibody | Rabbit | WB (1:5000) | Cell Signaling Technology | Cat# 7074 |
| Anti-XBP1 antibody | Mouse, Rat, Human | WB (1:1000) | Santa Cruz Biotechnology | Cat# sc-8015 |

***Supplementary Table 2.*** Sequence of primers for RT-qPCR.

| **Name** | **Sequence (5’→3’)** | **Supplier** |
| --- | --- | --- |
| mouse-*Gapdh*-RT-F | CATGGCCTTCCGTGTTCCT | TSINGKE |
| mouse-*Gapdh*-RT-R | GCCTGCTTCACCACCTTCT | TSINGKE |
| mouse-*Mettl3*-RT-F | CTGGGCACTTGGATTTAAGGAA | TSINGKE |
| mouse-*Mettl3*-RT-R | TGAGAGGTGGTGTAGCAACTT | TSINGKE |
| mouse-*Manf*-RT-F | CATCAATGAGGTGTCGAAGCCC | TSINGKE |
| mouse-*Manf*-RT-R | CACTGTGCTCAGGTCAATCTGC | TSINGKE |
| mouse-*U1*-RT-F | GCCTTCAAGACTCTGTTCGTGG | TSINGKE |
| mouse-*U1*-RT-R | CTCGATGAAGGCATAACCACGG | TSINGKE |
| mouse-*Ern1*-RT-F | GGCTACCATTATCCTGAGCACC | TSINGKE |
| mouse-*Ern1*-RT-R | CTCCTTCTGGAACTGTTGGTGC | TSINGKE |
| mouse-*Xbp1t*-RT-F | TGAAAAACAGAGTAGCAGCGCAGA | TSINGKE |
| mouse-*Xbp1t-*RT-R | CCCAAGCGTGTTCTTAACTC | TSINGKE |
| mouse-*Xbp1s*-RT-F | GGTCTGCTGAGTCCGCAGCA | TSINGKE |
| mouse-*Xbp1s*-RT-R | AAGGGAGGCTGGTAAGGAAC | TSINGKE |
| mouse-*Xbp1u*-RT-F | CAGACTACGTGCACCTCTGC | TSINGKE |
| mouse-*Xbp1u*-RT-R | CAGGGTCCAACTTGTCCAGAAT | TSINGKE |
| mouse-*P4hb*-RT-F | CAACTGGCTGAAGAAACGCACG | TSINGKE |
| mouse-*P4hb*-RT-R | GAACTGCTTGGCAGAGTCTGAC | TSINGKE |
| mouse-*Dnajb9*-RT-F | AGCCATGAAGTACCACCCTGAC | TSINGKE |
| mouse-*Dnajb9*-RT-R | CGACTATTGGCATCCGAGAGTG | TSINGKE |
| mouse-*Eif2ak3*-RT-F | CCGATGTCAGTGACAACAGCTG | TSINGKE |
| mouse-*Eif2ak3*-RT-R | AAGACAACGCCAAAGCCACCAC | TSINGKE |
| mouse-*Atf4*-RT-F | AACCTCATGGGTTCTCCAGCGA | TSINGKE |
| mouse-*Atf4*-RT-R | CTCCAACATCCAATCTGTCCCG | TSINGKE |
| mouse-*Ddit3*-RT-F | GGAGGTCCTGTCCTCAGATGAA | TSINGKE |
| mouse-*Ddit3*-RT-R | GCTCCTCTGTCAGCCAAGCTAG | TSINGKE |
| mouse-*Atf6*-RT-F | GTCCAAAGCGAAGAGCTGTCTG | TSINGKE |
| mouse-*Atf6*-RT-R | AGAGATGCCTCCTCTGATTGGC | TSINGKE |

***Supplementary Table 3.*** Sequence of primers for m^6^A-RIP-qPCR.

| **Name** | **Sequence (5’→3’)** | **Supplier** |
| --- | --- | --- |
| mouse-*Manf*-F | GATGCCTAAATACGCCCCCA | TSINGKE |
| mouse-*Manf*-R | TGTCAGGAGCCCGCTTAAAAA | TSINGKE |
| mouse-*Gapdh*-F | CATGGCCTTCCGTGTTCCT | TSINGKE |
| mouse-*Gapdh*- R | GCCTGCTTCACCACCTTCT | TSINGKE |
